# Clinical characteristics of malignant germ cell tumors in adolescents: A multicenter 10‐year retrospective study in Beijing

**DOI:** 10.1002/cai2.87

**Published:** 2023-08-20

**Authors:** Qian Zhao, Miao Li, Qing Sun, Tian Zhi, Mei Jin, Wen Zhao, Xisi Wang, Chao Duan, Xiaoli Ma, Wanshui Wu, Weihong Zhao, Dongsheng Huang, Yan Su

**Affiliations:** ^1^ Medical Oncology Department Pediatric Oncology Center, Beijing Children's Hospital, Capital Medical University, National Center for Children's Health, Beijing Key Laboratory of Pediatric Hematology Oncology, Key Laboratory of Major Diseases in Children, Ministry of Education Beijing China; ^2^ Beijing Shijitan Hospital Capital Medical University Beijing China; ^3^ Peking University, First Hospital Beijing China; ^4^ Beijing Tongren Hospital Capital Medical University Beijing China

**Keywords:** adolescent, malignant germ cell tumors, prognosis, treatment

## Abstract

**Background:**

The aim of this study was to review clinical features of adolescent malignant germ cell tumors (MGCTs) in Beijing and analyze the peculiar characteristics of this age group.

**Methods:**

Clinical characteristics, pathological presentations, and survival outcomes of 34 patients were analyzed retrospectively.

**Results:**

Of 34 patients, 12 girls and 22 boys, 18 (52.9%) had an extra‐cranial tumor, including one testicular tumor, five ovarian tumors, one sacrococcygeal tumor, and 11 mediastinal tumors. Histologically, we found immature teratomas (*n* = 6), yolk sac tumors (*n* = 5), mixed malignant tumors (*n* = 5), an embryonic carcinoma (*n* = 1), and seminoma (*n* = 1). Three‐year event‐free survival (EFS) and overall survival (OS) were 48.8% and 62.9%, respectively. Another 16 (47.1%) patients had an intracranial tumor, including nine in the pineal region, five in the suprasellar region, one in basal ganglia, and one in cerebellopontine. All patients had localized disease and an excellent outcome with 3‐year EFS and OS of 93.7% and 100%, respectively.

**Conclusions:**

Adolescent MGCTs are rare with a strong dependence on gender, and the mediastina and pineal region are the most common tumor locations. The prognosis is promising compared with that of other adolescent tumors and MGCTs in other age groups. MGCTs in mediastina have a tendency to companion with other hematological malignancies, and the prognosis is extremely poor in these patients.

## Graphical abstract

Adolescent malignant germ cell tumors (MGCTs) are rare with a strong dependence on gender, and the mediastina and pineal region are the most common tumor locations according to extra and intracranial cranial region. The prognosis is promising compared with that of other adolescent tumors and MGCTs in other age groups. MGCTs in mediastina have a tendency to companion with other hematological malignancies, and the prognosis is extremely poor in these patients.

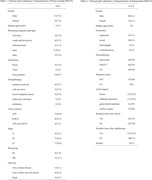

**Abbreviations**
AFP: alpha‐fetoprotein
CR: complete remission
MGCTs: malignant germ cell tumors
NGGCT: nongerminomatous germ cell tumors
β‐HCG: β‐human chorionic gonadotropin

## 1 INTRODUCTION

A malignant germ cell tumor (MGCT) arises from primordial germ cells, which migrate during embryogenesis from the yolk sac through the mesentery to gonads. It might primarily arise in the gonads (testicles and ovaries) and may also arise in the anterior mediastinum, central nervous system, and retroperitoneum. MGCTs are rare in childhood, representing only 3.5% of childhood cancers, but they are a common malignancy in adolescents [1]. The United Nations has formally defined adolescence as the period between 10 and 19 years of age [2], and patients in this age group have different biological behavior than other age groups [3]. The overall outcomes of patients treated for MGCTs are excellent. However, as seen in other cancers, outcomes for adolescent patients are significantly worse. This study focuses on the unique characteristics of patients with MGCTs in the adolescent group. We retrospectively analyzed clinical data and summarized the clinical characteristics, diagnosis, treatment status, and existing problems to provide evidence to support further clinical studies.

## 2 METHODS

### 2.1 Study participants

This study included newly diagnosed MGCT patients in adolescence between the ages of 10 and 19 from seven large children's diagnosis and treatment centers in Beijing, between January 1, 2010 and December 31, 2019. All children were followed up until June 30, 2020.

### 2.2 Treatment and outcome evaluation

Patients were diagnosed on the basis of the pathology of the primary tumor, tumor markers, and imaging. They were treated at a single center by multidisciplinary combined comprehensive treatment, mainly including surgery, chemotherapy, and radiotherapy. Systemic tumor imaging evaluation was performed to guide selection of surgical resection or direct neoadjuvant chemotherapy and to identify the tumor invasion site and any distant metastasis.

For extracranial MGCTs, systemic treatment mainly included surgical removal when possible, followed by chemotherapy if indicated. When initial surgery was not possible, neoadjuvant chemotherapy was necessary, followed by surgical removal of the residual tumor. Patients were stratified into three risk groups and four stages in accordance with the Children's Oncology Group classification [4]. Patients in the various risk groups underwent different chemotherapy regimens. Most patients in the intermediate risk (IR) group received the PEB regimen (etoposide, bleomycin, and cisplatin) [5], and underwent the JEB regimen (etoposide, bleomycin, and carboplatin) in only one hospital [6]. Patients in the high‐risk (HR) group received the C‐PEB regimen (etoposide, bleomycin, cisplatin, and cyclophosphamide) [7]. Chemotherapy was repeated at 21 days, and the number of courses was three to five cycles post‐surgery if evaluation indicated completed remission, including no residual disease on imaging and normalization of the alpha‐fetoprotein (AFP) level for more than three to four cycles.

For intracranial MGCTs, systemic treatment mainly included chemotherapy and radiotherapy. In such cases, a diagnostic biopsy may not be necessary and aggressive resection leading to morbidity is not the goal of surgical intervention, because these tumors are highly sensitive to chemotherapy and radiotherapy [8]. Chemotherapy permits lowering the dose of irradiation, thereby reducing toxicity [9]. Platinum‐based chemotherapy is quite effective. Chemotherapy alone is associated with a high risk of relapse [10].

### 2.3 Follow‐up and statistical analysis

Patients who stopped treatment without disease deterioration were withdrawn from treatment. Patients who terminated treatment because of disease deterioration were included in the statistical analysis. The most recent follow‐up time and disease status were the endpoint. Statistical endpoints were complete remission, progression, recurrence, and death due to disease. SPSS version 17.0 (SPSS, Inc.) was used to analyze data. Overall survival (OS) was defined as the time from admission to death or loss to follow‐up for any reason. Event‐free survival (EFS) was defined as the time from admission to initial tumor recurrence/metastasis, death, or loss to follow‐up for any reason. Data with normal distributions are expressed as the mean ± standard deviation. Data with nonnormal distributions are expressed as the median (range). Survival data were subjected to Kaplan‐Meier survival analysis. *p* < 0.05 was considered statistically significant.

## 3 RESULTS

### 3.1 Patient characteristics

Thirty‐four patients were recruited in our research. Of the 34 patients, 22 (64.7%) were boys and 12 (35.3%) were girls. The median age was 155.9 months (range, 121–201 months). Sixteen patients had intracranial tumors, and 18 patients had extra cranial tumors, including one in a testicle, five in ovaries, 11 in mediastinum, and one in the pelvic cavity.

### 3.2 Extracranial MGCTs

#### 3.2.1 Clinical information

Eighteen patients older than 10 years with newly diagnosed extracranial GCTs were recruited in this study, including 13 boys and five girls. Six (33.3%) patients had a gonadal tumor (one testicular and five ovarian), and 12 (66.7%) patients an extragonadal tumor (one sacrococcygeal and 11 mediastina). All patients presented with altered general conditions and abdominal or chest symptoms: chest pain (*n* = 5), cough and hemoptysis (*n* = 6), abdominal pain (*n* = 2), and a mass (*n* = 7) (Table 1).

**Table 1:** Clinical and laboratory characteristics of extracranial MGCTs.

	*n* (%)
Gender	
Male	13 (72.2)
Female	5 (27.8)
Median age (month)	155.9
Presenting symptoms and signs	
Chest pain	5 (27.8)
Cough and hemoptysis	6 (33.3)
Abdominal pain	2 (11.1)
Mass	7 (38.9)
Other	5 (27.8)
Localization	
Ovary	5 (27.8)
Testis	1 (5.6)
Extra gonadal	12 (66.7)
Histopathology	
Immature teratoma	6 (33.3)
Yolk sac tumor	5 (27.8)
Mixed malignant tumors	5 (27.8)
Embryonic carcinoma	1 (5.6)
Seminoma	1 (5.6)
Tumor markers	
AFP	17 (94.4)
β‐HCG	6 (33.3)
AFP and β‐HCG	6 (33.3)
Stage	
II	4 (22.2)
III	7 (38.9)
IV	7 (38.9)
Risk group	
IR	5 (27.8)
HR	13 (72.2)
Outcome	
Alive without disease	11 (61.1)
Lost to follow‐up with disease	4 (22.2)
Dead	3 (16.7)

Abbreviations: AFP, alpha–fetoprotein; β‐HCG, β‐human chorionic gonadotropin; HR, high‐risk; IR, intermediate risk; MGCT, malignant germ cell tumors.

Serum AFP was increased (43.24–90,474 ng/mL) in 17 patients, and a normal level (3.8 ng/mL) was found in one patient, whose primary tumor was located in the mediastina. β‐Human chorionic gonadotropin (β‑HCG) was elevated in six patients (2.88–5000 UI/mL).

On computed tomography scans, the maximum diameter of tumors ranged from 2.5 to 21.4 cm. Ten patients had a tumor maximum diameter of ≥10 cm. Metastases were diagnosed in seven patients (lymph node = 3, bone = 3, lung = 2, liver = 1, renal = 1, and pleura = 1). Four patients out of 18 (22.2%) had stage II, seven (38.9%) had stage III, seven (38.9%) had stage IV disease. Five were IR (27.8%), and 13 were HR (72.2%). No stage I or low‐risk patients were included in this study.

#### 3.2.2 Histopathology

Histologically, immature teratomas were found most frequently (*n* = 6), followed by yolk sac tumors (*n* = 5), mixed malignant tumors (*n* = 5), embryonic carcinoma (*n* = 1), and seminoma (*n* = 1).

#### 3.2.3 Treatment

Treatment options for adolescents with extracranial MGCTs were based on the primary site, histological type, and clinical condition. For 18 patients, treatment mainly included surgery and platinum‐based chemotherapy. Ten patients received surgery first, followed by chemotherapy, 7 received a diagnostic tumor biopsy and preoperative chemotherapy, followed by definitive tumor resection, and one was clinically diagnosed by imaging and tumor markers, who received a resection after neoadjuvant chemotherapy. Among them, the patients received a second surgery after chemotherapy for the residual tumor. Five IR patients received four to five cycles of chemotherapy, and 13 HR patients received six to eight cycles. One recurrent patient received high‐dose chemotherapy and hematopoietic stem cell rescue. For another recurrent patient, who was unsatisfied with chemotherapy, local radiation was recommended, but the patient abandoned the treatment because of tumor progression.

#### 3.2.4 Results and survival

Eleven (61.1%) patients were free of disease at a median follow‐up of 25 months (range, 5.5–77 months). Three‐year EFS and OS were 48.8% and 62.9%, respectively. Two patients were initially assessed as IR. Two cycles of chemotherapy were administered in accordance with the IR group regimen, and the tumor progressed in accordance with the evaluation. Then, chemotherapy was upgraded to the HR group, accompanied by surgery. Both of them achieved complete remission (CR) after four to five cycles of C‐PEB. The 11 patients were followed up in the outpatient clinic, and no relapse occurred.

Four (22.2%) patients were lost to follow‐up at a median follow‐up of 13 months (range, 1.5–37 months). Among them, one patient was lost to follow‐up after one cycle of chemotherapy and surgery. One patient underwent CR after standard treatment, and lost follow‐up after 37 months. One patient lost follow‐up after six cycles of chemotherapy because of progression to distant lymph node metastasis. The last patient was lost to follow‐up because of pelvic progression after standard treatment and hematopoietic stem cell rescue. The median time for tumor progression was 5 months.

Two patients progressed with acute myelogenous leukemia at 6 and 33 months from diagnosis, and died of multiple organ dysfunction at 2 months and 3 days after treatment of acute myelogenous leukemia, respectively. Another patient died of progression.

### 3.3 Intracranial MGCTs

#### 3.3.1 Clinical information

Sixteen patients were diagnosed with intracranial MGCTs, nine boys and seven girls with a median age of 152 months (range, 121–201 months). The most common complaints were headache (*n* = 8, 50%), nausea and vomiting (*n* = 6, 37.5%), endocrine‐related symptoms (*n* = 3, 18.8%), neurological symptoms, such as an unsteady gait and weakness of limbs (*n* = 2, 12.5%), and symptoms related to vision or hearing (*n* = 2, 12.5%).

Tumors were located in the pineal region in nine patients (56.3%) and suprasellar region in five (31.3%). In 12.5% of all patients, tumors were found in rare locations: basal ganglia (*n* = 1) and cerebellopontine (*n* = 1). All patients had localized disease, and no metastasis was found in this study cohort (Table 2).

**Table 2:** Clinical and laboratory characteristics of intracranial MGCTs.

	*n* (%)
Gender	
Male	9 (56.3)
Female	7 (43.7)
Median age (month)	152
Localization	
Suprasellar	5 (31.3)
Pineal	9 (56.3)
Basal ganglia	1 (6.2)
Cerebellopontine	1 (6.2)
Histopathology	
Germinoma	4 (25.0)
NGGCT	4 (25.0)
NA	8 (50.0)
Metastatic status	
M 0	16 (100)
M +	0 (0)
Initial surgery	
Biopsy	2 (12.5)
Subtotal resection	2 (12.5)
Gross total resection	4 (25)
Without surgery	8 (50)
Residual tumor after chemo	
Yes	4 (25.0)
No	12 (75.0)
Residual tumor after radiotherapy	
Yes	2 (12.5)
No	14 (87.5)
Relapse	1 (6.2)

#### 3.3.2 Diagnosis and histopathology

The diagnosis was confirmed histopathologically in eight patients, and another eight were clinical diagnosed by radiological findings and tumor markers. Tumors were classified as germinoma and nongerminomatous germ cell tumors (NGGCT) by histopathology. Histopathology showed germinoma in four patients (50%) and NGGCT in another four (50%). The serum AFP level was high in three, β‐HCG in seven, and both markers in one patient. In two patients analyzed for cerebrospinal markers, β‑HCG was high in both.

#### 3.3.3 Treatment

Surgical intervention was performed in eight (50%) patients at diagnosis. Gross total resection was performed in four (25%) patients, subtotal resection in two (12.5%), and biopsy in two (12.5%), followed by two to four chemotherapy courses and radiotherapy. The remaining eight (50%) patients were clinically diagnosed by radiological findings and tumor markers. Three to seven chemotherapy courses were administered with radiotherapy. Overall, radiotherapy was administered to 15 patients and doses varied between 40 and 55 Gy (median, 46 Gy).

#### 3.3.4 Results and survival

The median follow‐up time was 22.5 months. Relapsed disease was observed in one patient within 6 months. The relapse locations were the primary site. Residual tumor was found in four patients after completing chemotherapy (germinoma = 1; NGGCT = 3) and two after radiotherapy (germinoma = 0; NGGCT = 2). One NGGCT had γknife as part of the treatment strategy. Because all patients were localized, this study group had an excellent outcome with 3‐year EFS and OS of 93.7% and 100%, respectively.

## 4 DISCUSSION

Fonseca et al. [1] reported that MGCTs account for 13.9% of neoplasms between 15 and 19 years of age, and that their occurrence is higher in adolescents between 10 and 19 years of age. In our multicenter data, MGCT patients accounted for 8.2% (34/411) of all adolescent cancer patients, which was lower than in Fonseca et al.'s study. Genetic variation may be a risk factor in Han Chinese populations, and some patients were treated in adult hospitals, which was the main reason for the relatively small number of adolescent MGCTs.

In terms of the gender distribution, trend analysis in Latin America [11] (1988–2007) considered the average incidence in adolescent and young adult cancer cases, of which 46.30% occurred in males and 53.69% affected females. It has been reported that 91% of MGCTs in adolescents and young adults occur in males [12]. In our study, there were 22 (65%) males compared with 12 (35%) females, indicating that MGCTs are more common in males regarding adolescent and young adult cancers. Wong et al. [9] found a male:female ratio of 82:32 for intracranial adolescent MGCTs. In the United States [13, 14], the maximum incidence rate of extracranial MGCTs is 11.4 per 100,000 in males, but only one per 100,000 in females, indicating that the number of male patients is higher than that of females, which was consistent with our study.

Extracranial MGCTs are neoplasms that occur infrequently, representing 2%–3% of cancers in this group [15]. Extracranial MGCTs can present in both gonadal and extragonadal sites. In general, 10%–15% of MGCTs diagnosed in adolescents are in extragonadal locations [16]. However, in our study, 12 (66.7%) of 18 patients had extragonadal tumors, which conflicts with the results of previous studies. Mediastinum was the most common primary tumor site of extracranial MGCTs in our study. The overall outcomes of patients treated for MGCTs are excellent [17]. However, as seen in other cancers, outcomes of adolescent patients are significantly worse. Since the introduction of platinum‐based agents in chemotherapy regimens, the survival rates of children with extracranial MGCTs ranges from 75% to >90% [18]. However, adolescent patients experience statistically significantly worse EFS than children or adults [19]. Cost et al. [20] examined EFS of males with testicular cancer across three age groups. They found that adolescent patients had the lowest 3‐year EFS at 60%, which was significantly worse than in children (defined as ages younger than 13 years; EFS, 87%) and adults (ages older than 19 years; EFS, 80%). In a large population‐based study in Germany, the 5‐year survival rate was 92% for extracranial MGCTs [21]. In our study, 3‐year EFS and OS were 48.8% and 62.9%, respectively, which was lower than that of other studies and could be partially explained by the high number of patients with advanced disease.

In our study, two patients with mediastina MGCTs had acute myelogenous leukemia at 6 and 33 months from diagnosis. Primary MGCT of the mediastinum has a tendency of hematological malignancies, and the risk of associated hematological malignancies is highest in the first year after diagnosis. There is no specific clinical or biological index to predict the occurrence of hematological malignancies. There is no guideline for the treatment of hematological malignancies in patients with mediastina MGCTs. Compared with hematological tumor cells from bone marrow, hematological malignant tumor cells from MGCTs respond poorly to chemotherapy, which may be due to their different genetic mechanisms. The prognosis is extremely poor, and these patients should be treated as acute leukemia with poor prognosis and receive hematopoietic stem cell transplantation as soon as possible.

Intracranial MGCTs are a rare heterogeneous group of tumors. The age‐standardized incidence rate for 0–19 years of age varies by country between 0.1/1,000,000 and 3.8/1,000,000 [22]. The incidence rate in East Asian countries is higher than that in the United States and other Western countries [23, 24]. The peak incidence for intracranial MGCTs is 10–14 years of age. Kurucu et al. [25] showed that most cases (51.9%) were between 10 and 14 years of age with a median of 11.5 years. In the United States, 60%–80% of all intracranial MGCTs are located in the pineal region, while this ratio is 40%–44% in Japanese registries. Pineal tumors comprised 56.3% of 16 cases in our study, which was similar to other studies. In another study, approximately two‐thirds of cases were germinomas, whereas in our study, germinomas and NGGCT were seen at approximately equal frequencies. Treatment of intracranial MGCTs requires a multidisciplinary approach. Aggressive resection leading to morbidity is not the goal of surgical intervention, because these tumors are highly sensitive to chemotherapy and radiotherapy. A study from Hacettepe University [25] reported that 5‐year OS and EFS of intracranial MGCTs were 72.6% and 57.2%, respectively. In our study, 3‐year EFS and OS were 93.7% and 100%, respectively, because no patients had metastasis and the prognosis was better than in the other report [24, 25].

## 5 CONCLUSIONS

Adolescent MGCTs are rare with a strong dependence on gender. The prognosis is promising compared with that of other adolescent tumors and MGCTs in other age groups. There were some limitations in our study. The retrospective analyzes with a long time frame made it difficult to interpret the results. The diagnostic methods, treatment modalities, surgical techniques, and supportive therapies differed regarding time and hospital. Additionally, systematic management of tumor therapy is insufficient. Follow‐up management focuses on tumor stability alone. In the retrospective analysis, there was a lack of information regarding chemotherapeutic drug toxicity, growth and development disorders, and long‐term organ functions, which should be further summarized.

## AUTHOR CONTRIBUTIONS

**Qian Zhao**: writing manuscript. **Qian Zhao**, **Miao Li**, **Qing Sun**, and **Tian Zhi**: collected clinical data. **Xiaoli Ma**: conceived idea and managed cases. All authors were involved in drafting, reviewing, and approved the final version for submission.

## CONFLICT OF INTEREST STATEMENT

The authors declare no conflicts of interest.

## ETHICS STATEMENT

This study was a multicenter retrospective clinical study that was registered in the National Medical Research Registration Information System, reviewed, and approved by the Ethics Committee of Beijing Children's Hospital, Capital Medical University (Approval number: 2020‐Z‐012).

## INFORMED CONSENT

Written informed consent was obtained from the parents and their patients for the study and the publication of the article and all information contained within. Copies of the written consent are available for review by the Editor of this journal.

## Data Availability

All data generated or analyzed during this study are included in this published article.
